# Screening and identification of potential prognostic biomarkers in metastatic skin cutaneous melanoma by bioinformatics analysis

**DOI:** 10.1111/jcmm.15822

**Published:** 2020-09-01

**Authors:** Zufeng Sheng, Wei Han, Biao Huang, Guoliang Shen

**Affiliations:** ^1^ Department of Burn and Plastic Surgery The First Affiliated Hospital of Soochow University Suzhou China; ^2^ Department of Surgery Soochow University Suzhou China

**Keywords:** bioinformatics analysis, biomarker, metastatic melanoma, primary melanoma, prognosis

## Abstract

Skin cutaneous melanoma (SKCM) is a multifactorial disease that presents a poor prognosis due to its rapid progression towards metastasis. This study focused on the identification of prognostic differentially expressed genes (DEGs) between primary and metastatic SKCM. DEGs were obtained using three chip data sets from the Gene Expression Omnibus database. The protein‐protein interaction network was described by STRING and Cytoscape. Kaplan‐Meier curves were implemented to evaluate survival benefits within distinct groups. A total of 258 DEGs were distinguished as possible candidate biomarkers. Besides, survival curves indicated that *DSG3*, *DSC3*, *PKP1*, *EVPL*, *IVL*, *FLG*, *SPRR1A* and *SPRR1B* were of significant value to predict the metastatic transformation of melanoma. To further validate our hypotheses, functional enrichment and significant pathways of the hub genes were performed to indicate that the most involved considerable path. In summary, this study identified substantial DEGs participating in melanoma metastasis. *DGS3*, *DSC3*, *PKP1*, *EVPL*, *IVL*, *FLG*, *SPRR1A* and *SPRR1B* may be considered as new biomarkers in the therapeutics of metastatic melanoma, which might help us predict the potential metastatic capability of SKCM patients, thus provide earlier precautionary treatments. However, further experiments are still required to support the specific mechanisms of these hub genes.

## 1 INTRODUCTION

Skin cutaneous melanoma (SKCM) is the deadliest cancer among commonly encountered skin malignant tumours due to its extreme aggressiveness and dissemination.^1^ At present, SKCM is usually diagnosed in the late grades of metastatic tumours, which could drive patients to a poor response to the therapeutic strategies.^2^ Therefore, we need to explore the potential biomarkers and therapeutic targets to improve the diagnosis and therapy of invasive melanoma.

The malignant transformation of melanocytes is a multistep process. Melanocytes change their characteristics throughout the process, which enables them to proliferate and migrate.^2^ Numerous methods such as assessing excised tumours, utilization of biomarkers and imaging techniques had been applied to detect and monitor patients with SKCM.^1^ The currently known histopathologic features such as tumour thickness and ulceration status have been used for melanoma detection and prognosis prediction.^3^ However, the inevitable biases in the measurements of these features affect their application in evaluating melanoma prognosis. In the last few years, there has been a rising interest in the bioinformatics analysis, which can be applied to illustrate large and complicated data sets associated with various cancers. In this study, we screened out differentially expressed genes (DEGs) between primary and metastatic melanoma tissue and utilized bioinformatics analysis to distinguish hub genes and a range of functional enrichment. We are trying to identify the signatures of gene expression that associate with metastasis and survival in melanoma and find out more effective metastasis‐associated biomarkers to achieve precision medicine.

## 2 METHODS

### 2.1 Data collection and DEGs screening

DEGs were obtained from three chip data sets on the Gene Expression Omnibus database by using GEO2R (detailed in the Methods S1).

### 2.2 Functional enrichment analysis of DEGs

The GO and KEGG pathway analyses of DEGs were performed using Database for Annotation, Visualization and Integrated Discovery (detailed in the Methods S1).

### 2.3 Construction of PPI network and identification of hub gene

A protein‐protein interaction network was drawn with the Search Tool for the Retrieval of Interacting Genes/Proteins (STRING) to distinguish the hub genes and explore the interplays among the DEGs (detailed in the Methods S1).

### 2.4 Validation of hub genes

To further screen the significant hub genes, GraphPad Prism software was utilized to illustrate the differential expression of 369 metastatic melanoma and 103 primary melanoma samples from TCGA database (detailed in the Methods S1).

### 2.5 Kaplan‐Meier survival analysis

Kaplan‐Meier analyses were performed in GraphPad Prism software to investigate the correlation between the hub genes expression and the overall survival of patients with SKCM (detailed in the Methods S1).

### 2.6 Hub genes analysis

See details in the Methods S1.

### 2.7 Transcription factor network

See details in the Methods S1.

## 3 RESULTS

### 3.1 Hub genes screening between primary and metastatic melanoma tissue

**Figure 1:**
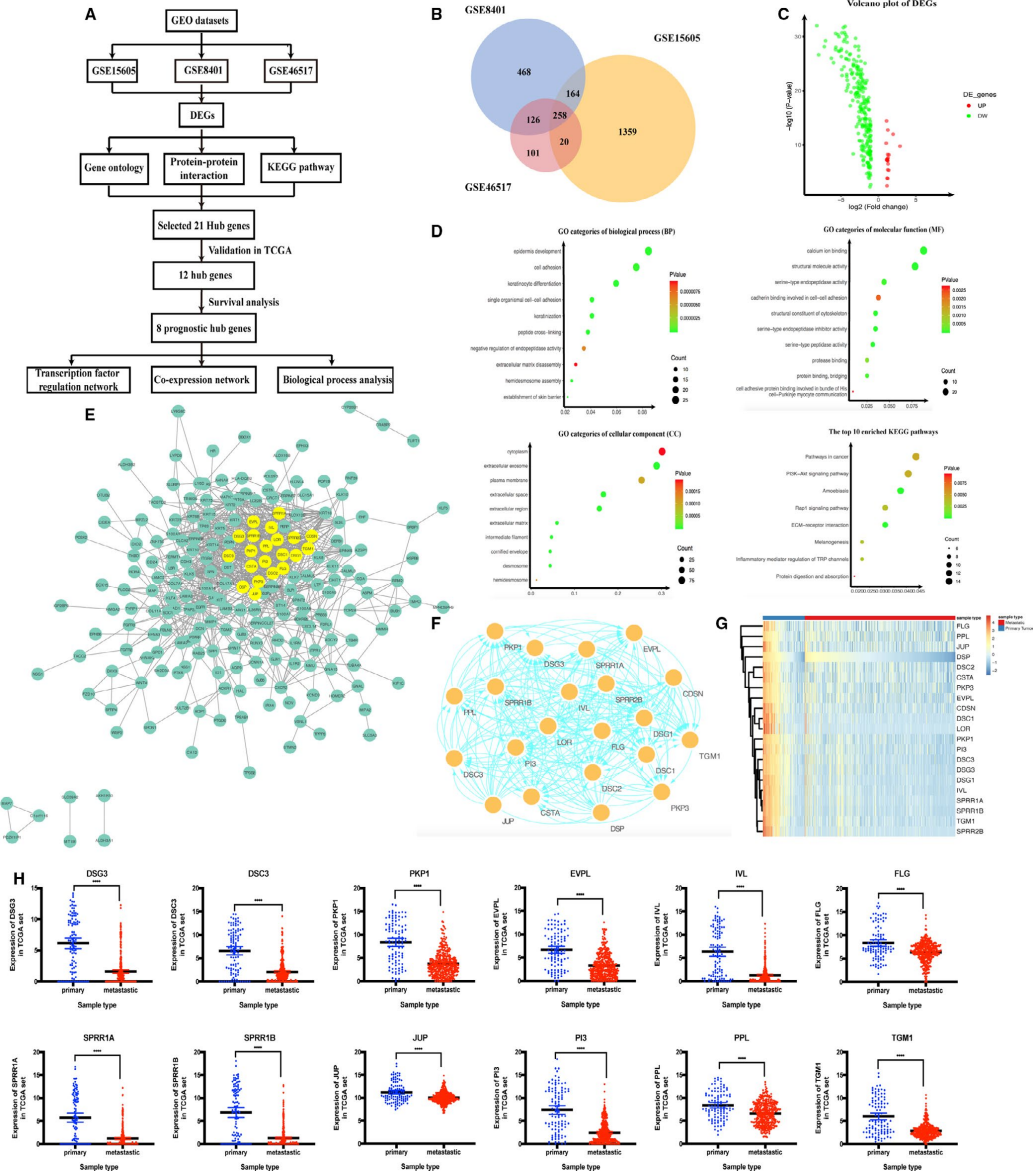
A, Flowchart of bioinformatics analysis. B‐C, Venn diagram, volcano plot and functional enrichment analysis of DEGs. D, The top 10 enriched GO categories of biological process (BP), molecular function (MF), cellular component (CC) and KEGG pathways. E, Protein‐protein interaction network of the differentially expressed genes (DEGs). F, The module obtained from protein‐protein interaction network with the highest score. A sum of 21 DEGs is involved in the module. G, Hierarchical partitioning of 21 DEGs on the basis of mRNA microarrays. H, Validation of the hub genes in TCGA. The expression of the hub genes comes from 369 metastatic and 103 primary melanoma samples. *P*‐value < .05 was regarded statistically significant. Metastatic tissues were drawn in red and primary tissues in blue

Among the three data sets, 258 genes were overlapped between primary and metastatic SKCM (Figure 1B,C). The top 10 of the most significant results for functional enrichment were presented, respectively, in Figure 1D. Using the STRING online database and the MCODE plug‐in from Cytoscape, a sum of 21 nodes and 209 edges were clustered into the PPI network complex (Figure 1E‐F). Heat map, based on TCGA cohort, showed that potential co‐expression relationships between primary and metastatic SKCM might be found in the 21 hub genes (Figure 1G).

### 3.2 Clinicopathological statistical analysis and survival outcomes

**Figure 2:**
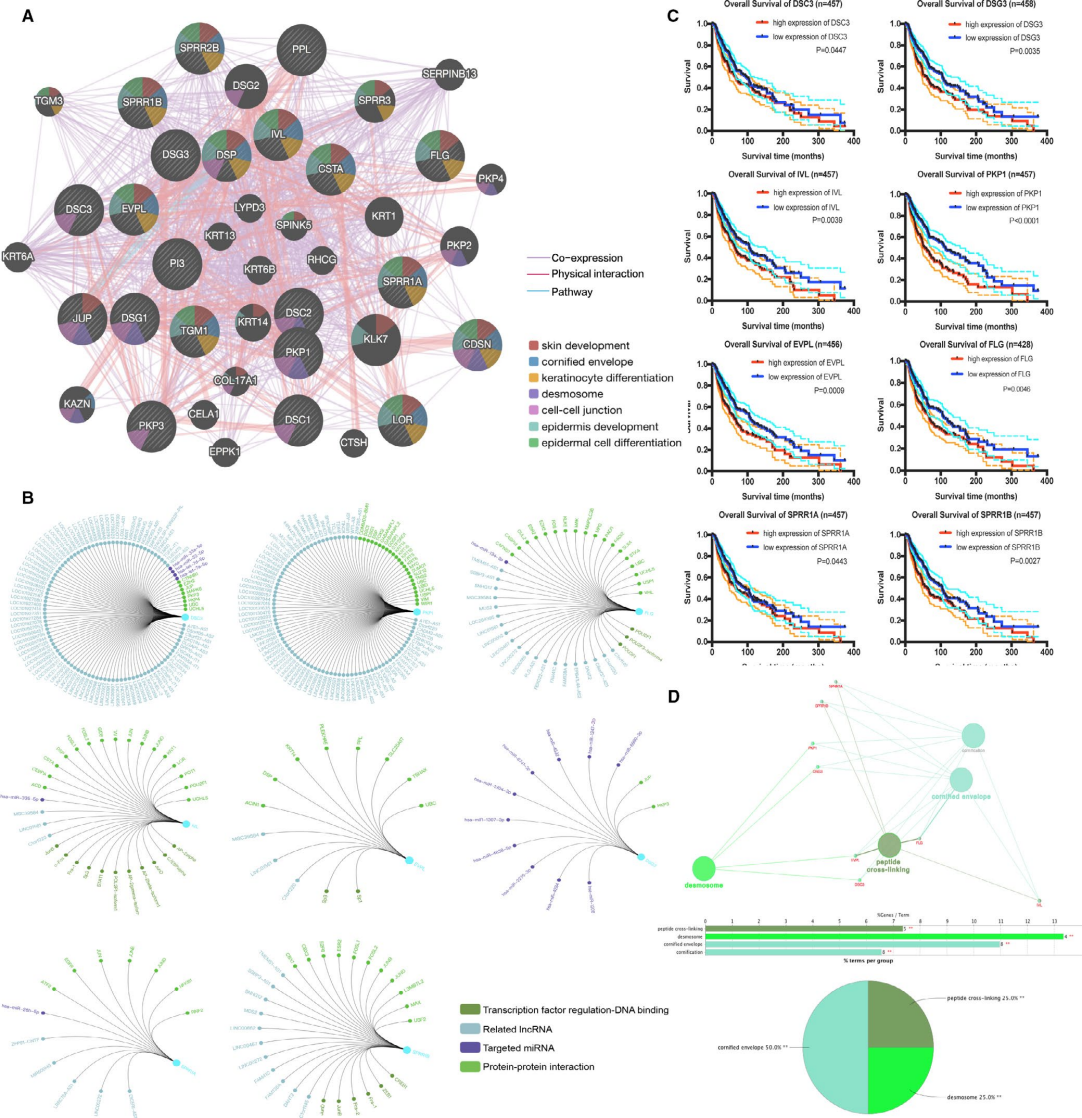
A, The co‐expression network was constructed with eight DEGs that were screened out from metastatic melanoma vs primary melanoma. B, Transcription factor regulation network was constructed in *DSC3*, *PKP1*, *FLG*, *IVL*, *EVPL*, *DSG3*, *SPRR1A* and *SPRR1B*. Different colour nodes and line represented different regulator functions. C, Overall survival curves of the hub genes. Each elevated expression in *DSC3*, *DSG3*, *EVPL*, *FLG*, *IVL*, *PKP1*, *SPRR1A* and *SPRR1B* hub genes displayed considerably significant poor OS in SKCM samples. *P* < .05 was considered statistically significant. D, Biological process analysis of the hub genes. Different colours of nodes refer to the different functional annotation of ontologies. Adjusted *P*‐value < .05 was regarded as the threshold

Based on the TCGA database, the expression of twelve hub genes was higher in primary tissues than in metastatic tissues (*P* < .05, Figure 1H), and the other nine genes showed no significant difference. After screening the more relevant hub genes, we conducted the survival analysis of the hub gene by using the Kaplan‐Meier curves. The outcomes revealed that overexpression of *DSG3*, *DSC3*, *PKP1*, *EVPL*, *IVL*, *FLG*, *SPRR1A* and *SPRR1B* genes predicted worse OS (*P* < .05) in SKCM patients (Figure 2C).

### 3.3 Co‐expression network analysis and transcription factor network

Co‐expression network analysis for the eight hub genes showed the relevant expression genes, physical interactions and pathways and also performed the main functions of each hub gene in cancer with a different colour in the node (Figure 2A). Subsequently, transcriptional regulation networks among the eight hub genes were displayed in Figure 2B. GO: BP and CC functional annotations were displayed in Figure 2D, with detailed function annotations listed in pie charts.

## 4 DISCUSSIONS

In the present study, eight hub genes, desmoglein 3 (*DGS3*), Desmocollin 3 (*DSC3*), plakophilin 1 (*PKP1*), envoplakin (*EVPL*), involucrin (*IVL*), filaggrin (*FLG*), small proline‐rich protein 1 A (*SPRR1A*) and small proline‐rich protein 1 B (*SPRR1B*), were considered to be significantly associated with the prognosis of patients with SKCM. The previous research demonstrated that these genes performed essential roles in the processes of epidermal development, keratinocyte differentiation, cell‐to‐cell signalling and cell adhesion.^4^ The disruptions of these processes may foster primary melanoma switch into invasive, active melanoma.

The function of *DSG3*, *DSC3*, *PKP1* and *EVPL* is associated with desmosomes, which are active intercellular junctions that are vital for cell cohesion and tissue integrity. *DSC3* and *DSG3* are both desmosomal cadherin that attaches the intermediate filaments of neighbouring cells and award stable cell adhesion.^5^, ^6^, ^7^ A recent study confirmed that disruption of cell adhesion might induce uncontrolled cell proliferation and played a crucial role in epithelial to mesenchymal transition (EMT), cancer formation, progression and tumour propagation.^5^ EMT is a fundamental phenomenon during embryonic morphogenesis, and it can enhance cellular mobility.^8^ When tumour cells abnormally reactivate EMT, they will gain the capability of invasion and dissemination to distant organs. Similar processes may arise in melanoma carcinogenesis. Feng et al^9^ suggested that the lower expression of *DSC3* and *DSG3* in malignant tissue was observed compared to the standard oral epithelium. However, other researchers revealed that *DSG3* was overexpressed in squamous cell lung cancer.^10^ *DSG3* seemed to be a pleiotropic gene that can influence both cell‐cell adhesion and cell movement, depending on the microenvironment circumstances in the process. *DSG3* may promote cell‐cell adhesion in healthy epithelial cells and raise its carcinogenic activity in mutated cells where the DSM structure is damaged.^5^ *PKP1* is a member of the armadillo protein family which enhances the interaction of desmosomes with the cytoskeleton, thereby strengthening desmosomal adhesion.^11^ *EVPL* is a member of the plakin family of proteins that forms an element of desmosomes and the epidermal cornified envelope.^12^ Hu N et al showed that *EVPL* was lower‐expressed in esophageal squamous cell carcinoma (ESCC) compared to healthy tissue, and it may be applied for early detection of ESCC.^13^ However, the function of desmosomes in the progression of SKCM remains unclear.

The cornified envelope of the skin is a sizeable insoluble polymer composed by cross‐linking of several protein precursors, including *IVL*, keratolinin, *FLG* and loricrin. *SPRR1* gene encodes a precursor of the keratinocyte cornified envelope, which displays in terminally differentiating human keratinocytes. *FLG* located on chromosomal locus 1q21.3, which is a reported susceptibility site in SKCM. Other genes on 1q21.3 code for proteins also focus on the terminal differentiation of keratinocytes.^14^ They present a crucial role in establishing and maintaining the epidermal barrier. Disruption of the integrity and stability of the epidermal barrier was a hallmark of cancer. Filaggrin can be degraded into free amino acids, which produce the natural moisturizing factor of the epidermis. Decreased variation in *FLG* was a significant risk factor for atopic dermatitis. Loss of function in *FLG* was assumed to enhance the susceptibility of skin malignancies due to reduced levels of its degradation products, urocanic acid, which may be protective towards ultraviolet irradiation.^15^ However, few findings were focusing on the value of these genes in melanoma metastasis. Our present findings will encourage further investigations of the clinical significance of hub genes in metastatic SKCM.

In summary, this study identified significant DEGs participating in melanoma metastasis. Down‐regulated genes, including *DGS3*, *DSC3*, *PKP1*, *EVPL*, *IVL*, *FLG*, *SPRR1A* and *SPRR1B*, may be considered as new biomarkers in the therapeutics of metastatic melanoma, which might help us predict the potential metastatic capability of SKCM patients, thus provide earlier precautionary treatments. However, further experiments are still required to support the specific mechanisms of these hub genes.

## CONFLICT OF INTEREST

The authors declared that they have no conflicts of interest in this work.

## AUTHOR CONTRIBUTIONS

**Zufeng Sheng:** Conceptualization (equal); Data curation (lead); Formal analysis (lead); Funding acquisition (equal); Investigation (lead); Methodology (lead); Project administration (equal); Resources (lead); Software (lead); Supervision (equal); Validation (equal); Visualization (lead); Writing‐original draft (lead); Writing‐review & editing (lead). **Wei Han:** Conceptualization (equal); Data curation (equal); Formal analysis (equal); Investigation (equal); Methodology (equal); Project administration (equal); Resources (equal); Software (equal); Supervision (equal); Visualization (equal); Writing‐original draft (supporting); Writing‐review & editing (supporting). **Biao Huang:** Conceptualization (supporting); Data curation (supporting); Methodology (supporting); Resources (supporting); Software (supporting). **Guoliang Shen:** Conceptualization (equal); Formal analysis (supporting); Funding acquisition (lead); Investigation (lead); Project administration (lead); Supervision (supporting); Validation (equal); Writing‐original draft (supporting); Writing‐review & editing (supporting).

## Supporting information

Method S1

## Data Availability

The data that support the findings of this study are openly available in the Gene Expression Omnibus (GEO) database at https://www.ncbi.nlm.nih.gov/geo/ (reference number GSE46517, GSE15605 and GSE8401) and in The Cancer Genome Atlas (TCGA) database at https://genme-cancer.ucsc.edu/ (cohort: TCGA Melanoma IlluminaHiSeq, n = 474, TCGA Hub).
