# Supplementary material for: Screening and identification of potential prognostic biomarkers in metastatic skin cutaneous melanoma by bioinformatics analysis
Source: J Cell Mol Med. 2020 Sep 1;24(19):11613–8. doi: 10.1111/jcmm.15822 (PMC7576265; doi:10.1111/jcmm.15822)
Supplement: Supplementary file 1 — Method S1 [file JCMM-24-11613-s001.docx]

**2 METHODS**

**2.1 Data collection**

Three gene expression datasets (GSE46517, GSE15605, GSE8401) were acquired from the Gene Expression Omnibus (GEO) database (https://www.ncbi.nlm.nih.gov/geo/). GSE8401 includes the expression profile of 32 primary skin melanomas and 51 metastatic skin melanomas^1^, GSE46517 includes 31 primary skin melanomas and 73 metastatic skin melanomas^2^ and GSE15605 comprises 46 primary skin melanomas and 12 metastatic skin melanomas^3^.

**2.2 Inclusion criteria of the DEGs**

The DEGs between primary and metastatic melanoma samples were selected by GEO2R (https://www.ncbi.nlm.nih.gov/geo/geo2r/)，which is an online analytics platform that enables users to compare two or more groups of samples downloaded from GEO Series to identify regulatory genes following specific conditions. $|logFC|$ > 1 and p-value < 0.05 were regarded as the cutoff value for DEGs selecting. Afterwards, Venn charts of the genes were mapped for the three databases.

**2.3 Functional enrichment analysis of DEGs**

Gene Ontology (GO) analysis is a functional study tool of extensive transcriptomics data or genomic concerning three models: Molecular function (MF), cellular component (CC) and biological process (BP), while Kyoto Encyclopedia of Genes and Genomes (KEGG) pathway analysis offers information about how molecules or genes operate^4,5^. The GO and KEGG pathway analyses were performed using Database for Annotation, Visualization and Integrated Discovery (DAVID, https://david.ncifcrf.gov/)^6,7^. The p-value < 0.05 was regarded as a statistically significant threshold.

**2.4 Construction of PPI network and Identification of hub gene**

A protein-protein interaction network (PPI) was drawn with the Search Tool for the Retrieval of Interacting Genes/Proteins (STRING, https://string-db.org/)^8^ to distinguish the hub genes and explore the interplays among the DEGs. We set STRING score of 0.4 as the dependability threshold. The results were visualized by Cytoscape (version 3.7.2), which is a software platform for integrated analysis and visualization of a complex network. And then, the genes were imported into the Molecular Complex Detection (MCODE), which is a novel graph-theoretic clustering algorithm in Cytoscape, screening the hub genes with node score cut-off = 0.2, a degree cut-off = 2, k-core = 2, haircut on, and max. depth = 100.

**2.5 Validation of hub genes**

To further screen the significant hub genes, RNA-sequencing data of SKCM were downloaded from The Cancer Genome Atlas (TCGA) database (<https://genme-cancer.ucsc.edu/)>. GraphPad Prism software (Version 8.3.0) was utilized to illustrate the differential expression of 369 metastatic melanoma and 103 primary melanoma samples from TCGA database, and p < 0.05 was considered as statistical significance.

**2.6 Kaplan-Meier survival analysis**

Kaplan-Meier analyses were performed in GraphPad Prism software (Version 8.3.0) to investigate the correlation between the hub genes expression and the overall survival of patients with SKCM. We classified patients into low and high expression groups in accordance with the median level of each hub gene expression. We regarded the log-rank p-value < 0.05 as statistically significant and 95% confidence interval was also estimated.

**2.7 Hub genes analysis**

A network of the genes and their co-expression genes were investigated by GeneMANIA (http://genemania.org) online platform^9^, which explores multiple openly accessible biological datasets programmatically to find relevant genes. Also, GO: BP, CC, MF and KEGG functional enrichment of hub genes were analyzed and plotted using CluePedia (version 1.5.3) and ClueGO (version 2.5.3) ^10^.

**2.8 Transcription factor network**

Transcription factor regulation networks were constructed in *DGS3*, *DSC3*, *PKP1*, *EVPL, IVL*, *FLG*, *SPRR1A* and *SPRR1B* using R software (Version 3.3.2). We described significant nodes involved in co-regulation of *DGS3*, *DSC3*, *PKP1*, *EVPL*, *IVL*, *FLG*, *SPRR1A* and *SPRR1B* in circle plots (transcription factor regulation-DNA binding, transcription factor regulation-activation, related lncRNA, targeted miRNA and protein-protein interaction).

**REFERENCE**

1. Xu L, Shen SS, Hoshida Y, et al. Gene expression changes in an animal melanoma model correlate with aggressiveness of human melanoma metastases. *Mol Cancer Res.* 2008;6(5):760-769.

2. Kabbarah O NC, Feng B, Nazarian RM et al. Human melanoma samples comparing nevi and primary and metastatic melanoma. *PLoS One.* 2010;5(5):10770.

3. Raskin L FD, Giordano TJ, Thomas DG et al. Transcriptome profiling identifies HMGA2 as a biomarker of melanoma progression and prognosis. *J Invest Dermatol.* 2013;133(11):2585-2592.

4. Ashburner M, Ball CA, Blake JA, et al. Gene ontology: tool for the unification of biology. The Gene Ontology Consortium. *Nat Genet.* 2000;25(1):25-29.

5. Qiu T, Wang H, Wang Y, Zhang Y, Hui Q, Tao K. Identification of genes associated with melanoma metastasis. *Kaohsiung J Med Sci.* 2015;31(11):553-561.

6. Huang DW, Sherman BT, Tan Q, et al. The DAVID Gene Functional Classification Tool: a novel biological module-centric algorithm to functionally analyze large gene lists. *Genome Biol.* 2007;8(9):R183.

7. Zhou Z, Li Y, Hao H, et al. Screening Hub Genes as Prognostic Biomarkers of Hepatocellular Carcinoma by Bioinformatics Analysis. 2019;28(1_suppl):76S-86S.

8. Franceschini A, Szklarczyk D, Frankild S, Kuhn M, Research LJJJNA. STRING V9.1: Protein-Protein Interaction Networks, with Increased Coverage and Integration. 2012;41(D1).

9. David WF, Donaldson SL, Ovi C, et al. The GeneMANIA prediction server: biological network integration for gene prioritization and predicting gene function. 2010(suppl_2):suppl_2.

10. Bindea G, Galon J, Mlecnik B. CluePedia Cytoscape plugin: pathway insights using integrated experimental and in silico data. *Bioinformatics.* 2013;29(5):661-663.
